# Identification of microbiological factors associated with periodontal health disparities

**DOI:** 10.3389/fcimb.2023.1137067

**Published:** 2023-02-16

**Authors:** Bing-Yan Wang, Aize Cao, Meng-Hsuan Ho, Derek Wilus, Sally Sheng, Hsiu-Wan Meng, Elissa Guerra, Jianming Hong, Hua Xie

**Affiliations:** ^1^ School of Dentistry, University of Texas Health Science Center at Houston, Houston, TX, United States; ^2^ School of Applied Computational Sciences, Meharry Medical College, Nashville, TN, United States; ^3^ School of Dentistry, Meharry Medical College, Nashville, TN, United States; ^4^ School of Graduate Studies, Meharry Medical College, Nashville, TN, United States

**Keywords:** *Porphyromonas gingivalis*, periodontitis, health disparities, race, risk factor

## Abstract

The present study aimed at identifying risk factors associated with periodontitis development and periodontal health disparities with emphasis on differential oral microbiota. The prevalence of periodontitis is recently rising dentate adults in the US, which presents a challenge to oral health and overall health. The risk of developing periodontitis is higher in African Americans (AAs), and Hispanic Americans (HAs) than in Caucasian Americans (CAs). To identify potentially microbiological determinations of periodontal health disparities, we examined the distribution of several potentially beneficial and pathogenic bacteria in the oral cavities of AA, CA, and HA study participants. Dental plaque samples from 340 individuals with intact periodontium were collected prior to any dental treatment, and levels of some key oral bacteria were quantitated using qPCR, and the medical and dental histories of participants were obtained retrospectively from axiUm. Data were analyzed statistically using SAS 9.4, IBM SPSS version 28, and R/RStudio version 4.1.2. Amongst racial/ethnic groups: 1) neighborhood medium incomes were significantly higher in the CA participants than the AA and the HA participants; 2) levels of bleeding on probing (BOP) were higher in the AAs than in the CAs and HAs; 3) *Porphyromonas gingivalis* levels were higher in the HAs compared to that in the CAs; 4) most *P. gingivalis* detected in the AAs were the *fimA* genotype II strain that was significantly associated with higher BOP indexes along with the *fimA* type IV strain. Our results suggest that socioeconomic disadvantages, higher level of *P. gingivalis*, and specific types of *P. gingivalis* fimbriae, particularly type II FimA, contribute to risks for development of periodontitis and periodontal health disparities.

## Introduction

Periodontitis is one of the most widespread infectious diseases among adults, with an estimated 46% of dentate adults aged 30 years and older in the US suffering from some forms of the disease (Eke et al., 2015). Based on the data from the National Health and Nutrition Examination Surveys I, II, and III, periodontitis disproportionately affects different racial and ethnic groups. African Americans (AAs) and Hispanic Americans (HAs) experience higher incidences of periodontitis than Caucasian Americans (CAs) even after adjustment for all co-factors (Borrell et al., 2002; Thornton-Evans et al., 2013; Eke et al., 2020). Longitudinal studies on the natural history of periodontitis suggest that modifiable and non-modifiable risk factors potentially influence the onset, clinical presentation, and progression of the disease (Loe et al., 1978; Okamoto et al., 1988; Michalowicz et al., 1991; Grossi et al., 1994; Kornman et al., 1997; Borrell et al., 2004; Borrell and Talih, 2012; Genco and Borgnakke, 2013; Thornton-Evans et al., 2013). Smoking, age, and gender, as well as genetic and socioeconomic factors are well-known risk factors for periodontitis (Stashenko et al., 2011; Borrell and Crawford, 2012; Ioannidou, 2017; Naorungroj et al., 2017; Sutton et al., 2017). Additionally, the oral microbiome is a necessary, although insufficient, etiologic factor of periodontitis (Socransky and Haffajee, 2000; Jenkinson and Lamont, 2005). *Porphyromonas gingivalis*, one of the putative periodontal pathogens, has been well studied. *P. gingivalis* in the oral cavity, even in low abundance, can disrupt host-microbial homeostasis and elevate the virulence potential of oral microbial communities (Hajishengallis et al., 2011; Hajishengallis et al., 2012).

We previously reported an antagonistic relationship between *P. gingivalis* and *Streptococcus cristatus* (Wang et al., 2009). We found that *S. cristatus* can repress FimA expression in several *P. gingivalis* strains, including strains of *fimA* types I, II, and III (Xie et al., 2000; Xie et al., 2007; Wang et al., 2009). *S. cristatus* surface protein, arginine deiminase (ArcA), was identified as the signaling molecule to which *P. gingivalis* responds by repressing the expression of the *fimA* gene and consequently reducing production of fimbrial adhesin, FimA (Xie et al., 2007; Wu and Xie, 2010). We also discovered the expression of the *arcA* gene to be significantly higher in *S. cristatus* than in *Streptococcus gordonii*, suggesting that these two streptococcal species play distinct roles in the highly orchestrated dental plaque (Lin et al., 2008). Recently, we reported that the ratio of *S. cristatus* to *P. gingivalis* was significantly higher in CAs than in HAs and AAs with periodontitis (Wang et al., 2021). Based on these observations, we speculate that the interaction of specific bacteria may play an important role in periodontal health disparities. In the present work, we focused on levels of keystone pathogens and accessory bacteria and investigated a potential link between microbial compositions, particularly the prevalence of *P. gingivalis* and *S. cristatus*, and risks of periodontitis in AAs, CAs, and HAs with intact periodontium. Our results suggest that differences in the compositions of oral microbial communities may influence susceptibility to periodontitis.

## Materials and methods

### Study cohorts

The research protocol was approved by the Committee for the Protection of Human Subjects of the University of Texas Health Science Center at Houston (IRB number: HSC-DB-17-0636). Candidates were screened during routine dental visits at the clinic of the School of Dentistry, University of Texas Health Science Center at Houston between 2017 and 2022. Individuals aged 21-75 years with self-reported ethnicity/race of AAs, CAs, or HAs were enrolled after the initial periodontal examination that included determination of plaque index (PI), bleeding on probing (BOP) level, probing depth, and clinical attachment level on all teeth (Newman et al., 2018). Radiographs were taken during this screening phase to assess bone loss. The clinical oral examinations were performed by trained dental examiners who are faculty members of the School of Dentistry, University of Texas Health Science Center at Houston. The examiners are calibrated annually in the diagnosis of periodontitis. Based on the 2017 World Workshop classification (I.L. et al., 2018; Trombelli et al., 2018), all study participants diagnosed as periodontal health on an intact periodontium or biofilm-induced gingivitis on an intact periodontium met the following criteria: >24 teeth; no alveolar bone loss or clinical attachment loss; pocket depth ≤3mm (excluding pseudo pocket); no antibiotic therapy in the previous six months; and not pregnant. Information on demographics and self-reported dental and medical histories of the participants were abstracted from the Electronic Health Record. The socioeconomic statuses of these participants were determined by obtaining median incomes based on residential zip codes (https://www.incomebyzipcode.com/).

### Plaque sample collection

Dental plaque samples were collected by board-certified periodontists using sterile paper points at baseline prior to any dental treatment and labeled in numbers according to sampling sequences. The paper points were placed in the mesiobuccal sulci of the first molar in each quadrant for 1 minute and then immersed immediately in an Eppendorf tube containing 0.5 ml of Tris-EDTA (TE) buffer (pH 7.5) (Wang et al., 2009). Oral bacteria were harvested by centrifugation, and the bacterial pellet was resuspended in 100 µl TE buffer. Bacterial chromosomal DNA was released by two cycles of freezing at -80^°^C overnight and boiling for 10 minutes.

### Bacterial quantitation by qPCR

Oral bacteria including *P. gingivalis* and *S. cristatus*, were enumerated by qPCR, using the SYBR Green PCR mix (Bio-Red Laboratories Inc., Redmond, WA, USA) with species-specific primers listed in **Table 1**. *P. gingivalis* strains with different *fimA* genotypes were determined by PCR with strain-specific primers (Zheng et al., 2011). The level of total bacteria was determined using primers corresponding to the conserved sequences of cyanobacterial small subunit rRNA genes (Turner et al., 1999) (**Table 1**). Bacterial standard curves used to calculate the number of bacterial cells were prepared by quantifying known amounts of genomic DNA from each bacterial species using qPCR (Wang et al., 2009). Briefly, bacteria were grown in standard media, and optical density of bacteria was determined and adjusted to OD_600_=1.0 (containing approximately 10^9^ bacteria in one mL). After serials dilution, qPCR was conducted and corresponded to an OD. Bacterial ratios were obtained using numerical levels of two bacteria from the same sample. Bacterial quantitation by qPCR was performed by a designated technician without any knowledge of subject information. Clinical data abstraction and analysis were performed by different designated faculty members.

**Table 1:** Primers used in this study.

Gene	Primer sequences (5’-3’)
*Cyanobacterial 16S rRNA*	GGGCTACACACGYGCWAC GACGGGCGGTGTGTRCA
*S. cristatus arcA*	CTGACGAAGCGAAAGGTCTG ATGTGGTTGAGCGATACAGC
*S. gordonii 16S rRNA*	CCACACTGGGACTGAGACAC TGCTCGGTCAGACTTTCGTC
*P. gingivalis16s rRNA*	TGTAGATGACTGATGGTGAAA ACTGTTAGCAACTACCGATGT
*P. gingivalis fimA I*	CTGTGTGTTTATGGCAAACTTC AACCCCGCTCCCTGTATTCCGA
*P. gingivalis fimA Ib*	CTCTTAAGATCAAGCGTGTA TGTCAGATAATTAGCGTCTCG
*P. gingivalis fimA II*	AACCCCGCTCCCTGTATTCCGA ACAACTATACTTATGACAATGG
*P. gingivalis fimA III*	ATTACACCTACACAGGTGAGGC AACCCCGCTCCCTGTATTCCGA
*P. gingivalis fimA IV*	CTATTCAGGTGCTATTACCCAA AACCCCGCTCCCTGTATTCCGA
*P. gingivalis fimA V*	AACAACAGTCTCCTTGACAGTG TATTGGGGGTCGAACGTTACTGTC

### Statistical analysis

Statistical analysis was performed for each group. Continuous variables were assessed for normality and log10-transformed as needed. Two-sided statistical tests were performed using a 5% significance level. Comparison of demographics, dental and medical histories, and oral microbial profiles among AAs, CAs, and HAs of different ages was performed using one-way analysis of means (ANOVA) for continuous variables and the chi-square test or Fisher’s exact test for categorical variables. Ordinal logistic regression was used to measure the association between race/ethnicity and levels of *P. gingivalis* with adjustment for age. The SAS software (version 9.4; SAS Institute, Cary, NC), IBM SPSS (version 28) and R/RStudio version 4.1.2 were used to conduct all statistical analyses.

## Results

### Demographics and clinical characteristics of the study cohort

Three hundred and forty individuals with intact periodontium were enrolled, including 104 AAs, 115 CAs, and 121 HAs. No significant difference was found among these racial and ethnic groups with regards to age, gender, and total number of teeth. To investigate whether social inequalities exist among these groups, neighborhood median incomes based on the participates’ zip codes were obtained. As presented in **Table 2**, the neighborhood median income of the CA group was significantly higher ($72,000) than those of the AA and HA groups ($59,000 and $54,000, respectively) (*P*<0.001).

**Table 2:** Characteristics of the study cohort.

Racial/ethnic groups	AA	CA	HA	Total	*P* – value
Demographics					
Gender (N, M/F)	36/68	42/73	32/89	110/230	0.21
Age (year; median, IQR)	43 (31, 57)	42 (28, 58)	39 (29, 47)	40 (29, 55)	0.06
Zip code income ($×1,000; medium, IQR)	59 (45, 75)	72 (59, 100)	54 (42, 71)	62 (47, 80)	<0.001*
Dental history and evaluation					
BOP (median, IQR)*^a^*	28 (17, 47)	14 (6, 27)	19 (8, 35)	20 (9, 37)	0.001*
PI (median, IQR)*^b^*	52 (29, 73)	44 (26, 63)	42 (25, 61)	44 (26, 69)	0.17
Tooth number (median, IQR)*^c^*	27 (25, 29)	28 (26, 28)	28 (26, 28)	28 (26, 28)	0.40
Dry mouth (%)	17.3	18.3	8.3	49 (14.4)	0.06
TMJ disorders (%)	35.6	46.1	44.6	144 (42.4)	0.24
Medical history					
BMI (median, IQR)*^d^*	28 (25, 33)	26 (24, 30)	28 (24, 31)	27 (24, 31)	0.06
Diabetes (N, %)	7 (6.7)	5.0 (4.3)	3.0 (2.5)	15 (4.4)	0.30
Hematologic disorders (N, %)*^e^*	11 (10.6)	16 (13.9)	17 (14.0)	44 (12.9)	0.69
Arthritis (N, %)	12 (11.5)	16 (13.9)	9 (7.4)	37 (10.9)	0.27
Osteoporosis (N, %)	2 (1.9)	2 (1.7)	4 (3.3)	8 (2.4)	0.74
Cancers (N, %)	6 (5.8)	5 (4.3)	2 (1.7)	13 (3.8)	0.23
Allergy (N, %)	30 (28.8)	37 (32.2)	28 (23.1)	95 (27.9)	0.29
Tobacco user (N, %)	8 (7.7)	12 (10.4)	8 (6.6)	28 (8.2)	0.55

^a^BOP: Bleeding on probing, detailed BOP data is listed in **Supplemental Table 1**.

^b^PI: Modified O’Leary plaque index, detailed BOP data is listed in **Supplemental Table 1**.

^c^Tooth number is based on 32.

^d^BMI, Body mass index.

^e^Hematologic disorders including hemophilia A, hemophilia B, Von Willebrand, idiopathic thrombocytopenic purpura, medication-induced bleeding, and other bleeding disorders.

*Statistical significance among the racial/ethnic groups.

Significant difference BOP levels were found among the racial/ethnic groups of the study participants. The highest degree of BOP was observed in the AA group with an interquartile range (IQR) of 28, followed by that in the HA group (IQR of 19) and in the CA group (IQR of 14) (*p* < 0.001) (**Table 2**). The severities of BOP levels in these groups were further investigated. We found 39.1% of the CAs to have BOP level lower than 10% compared to 33.1% of HAs and 14.4% of the AAs (*P*-value <0.001) (**Table 3**). While, BOP levels greater than 30% was found in 48.1% of the AAs, 30.6% of the HAs, and only 20.9 of the CAs, indicating that levels of periodontal inflammation were more severe in the AA and HA participants. However, no significant differences were present in PI and incidences of some well-known risks of periodontitis including diabetes and tobacco use among the AAs, CAs, and HAs of this periodontal healthy cohort.

**Table 3:** Levels of BOP in different racial/ethnic groups.

BOP (%)	Racial/ethnic groups			*p*-value
	AA, N = 104	CA, N = 115	HA, N = 121	
<10	15 (14.4)	45 (39.1)	40 (33.1)	< 0.001
≥10 ≤30	39 (37.5)	46 (40.0)	44 (36.4)	
>30	50 (48.1)	24 (20.9)	37 (30.6)	

Levers of BOP were determined according to 2017 World Workshop classification (Newman et al., 2018; Wang et al., 2021). BOP <10% and ≥10% indicate clinical gingival health and gingivitis respectively. BOP ≥10% but ≤30% and BOP >30% indicate localized and generalized gingivitis, respectively.

### Distribution of oral bacteria in participants of different racial/ethnic groups

Levels of *P. gingivalis* and *S. cristatus* in the dental plaque samples were determined using qPCR with specific primers. *P. gingivalis* was detected in 23.5% of all participants (80/340). Detection rates of *P. gingivalis* were in 21.2% of the AAs, 18.2% of the CAs, and 30.6% of the HAs (**Table 4**), respectively; however, statistical significance was not reached. In addition, higher prevalence of *P. gingivalis* was found among younger participants (aged 21-35 years) of the AA (7.7%) and the HA (9.9%) groups than that in the CA group (4.3%), suggesting that AAs and HAs may be at risk for developing periodontitis at a younger age. Moreover, 68.2% of *P. gingivalis*-positive AA subjects (15/22) had greater than 10,000 *P. gingivalis* cells in their dental plaque samples, while 48.7% of *P. gingivalis*-positive HA subjects (18/37) had greater than 10,000 *P. gingivalis* cells in their plaque samples. In contrast, only 33% of *P. gingivalis*-positive CAs (7/21) had greater than 10,000 *P. gingivalis* cells in their plaque samples. The higher prevalence of *P. gingivalis* among younger participants (<35 years old) and higher levels of *P. gingivalis* in dental plaque samples from the AA and HA participants suggest a likelihood periodontal health disparity (**Table 4**). We further analyzed *P. gingivalis* levels across the racial/ethnic groups using an ordinal regression, with *P. gingivalis* level as a categorical outcome variable and age as a predictor. Compared to the CA participants, the HA participants had a significantly higher level of *P. gingivalis*, after adjusting for age (*P*-value = 0.019) with an adjusted odds ratio (AOR) of 2.413, indicating that HAs have a higher chance to carry a relatively more *P. gingivalis*. The AAs also exhibited higher levels of *P. gingivalis* compared to the CAs after adjusting for age [AOR = 1.812), though the difference was not statistically significant.

**Table 4:** Prevalence of *P. gingivalis* in participants of different ages and racial/ethnic groups.

Age (y)	Detection rates of *P. gingivalis* (%)											
	AA				CA				HA			
	I*^a^*	II	III	All	I	II	III	All	I	II	III	All
< 35	1.9	0.9	4.8	7.7	1.7	1.7	0.9	4.3	3.3	0.8	5.8	9.9
36-50	0	0	2.9	2.9	3.5	0.9	1.7	6.1	3.3	5	4.1	12.4
51-	0.9	2.9	6.7	10.5	1.7	2.6	3.5	7.8	2.5	0.8	5	8.3
Subtotal	2.9	3.9	14.4	21.2	7.0	5.2	6.0	18.2	9.1	6.6	14.9	30.6

^a^ Levels of P. gingivalis: I 1 to 1000, II, 1001 to 10,000, III >10,000 cells.

As *P. gingivalis* is classified into six *fimA* genotypes (types I and Ib to V) based on their nucleotide sequences of the *fimA* gene, the *fimA* genotype in each dental plaque sample was examined. The Detection rates of genotypes I, Ib, II, III, IV, and V were 5.6%, 3.8%, 5.0%, 4.4%, 2.6% and 2.1%, respectively. As shown in **Table 5**, no significant difference in the distribution of types I, Ib, III, IV, and V among the groups was detected. However, a significantly higher detection rate (50%) of type II in the AA group than in the CA (9.5%) and HA groups (10.8%) (*P*=0.009) was observed.

**Table 5:** Distribution of *P. gingivalis fimA* genotypes among different racial/ethnic groups.

Genotype of *fimA*	% of detection rate of *fimA* genotype				*p*-value
	AA, N = 22	CA, N = 21	HA, N = 21	Total	
I	13.6	19.1	32.4	23.8	0.053
Ib	4.6	23.8	18.1	16.3	0.135
II	50	9.5	10.8	21.3	0.009
III	13.6	14.3	24.3	18.6	0.158
IV	13.6	14.3	8.1	11	1.000
V	4.6	19	5.4	8.6	0.405

To test whether the presence of *P. gingivalis* impacts the abundance of microbial communities, we quantitated bacterial levels in the dental plaque samples using qPCR with specific primers of cyanobacterial *16S rRNA*. We found that 61.9 % of *P. gingivalis*-negative participants had fewer than 10^8^ total bacterial cells, whereas only 41.2% of *P. gingivalis*-positive participants had fewer than 10^8^ bacterial cells (**Table 6**). In other words, significantly more *P. gingivalis*-positive subjects had greater than 10^8^ total bacterial cells in their dental plaque samples (58.8%) than *P. gingivalis*-negative participants (*P* = 0.001). Association of FimA types with BOP and total bacterial levels was examined using ANOVA, which demonstrated that participants carrying *fimA* genotypes II or IV strain had higher BOP levels, followed by those with genotype I strain. Conversely, individuals carrying the *fimA* genotypes types Ib, III, and V strains exhibited significantly lower BOP levels (*P*=0.028) (**Table 7**). However, no significant association between BOP levels and total bacterial levels was observed. These results suggest an association between FimA types II and IV with periodontal inflammation and health disparity.

**Table 6:** Impact of *P. gingivalis* on bacterial biofilm formation.

Total bacterial level	Percentage of participants		
	Without *Pg*, N=260	With *Pg*, N=80	*p*-value
<10^8^ % (N)	61.9 (161)	41.2 (33)	0.001
>10^8^ % (N)	38.1 (99)	58.8 (47)	

**Table 7:** Correlation between the *fimA* genotypes and BOP levels or total bacteria numbers.

Characteristics	Genotype of *fimA*						
	I, N = 19	Ib, N = 13	II, N = 17	III, N = 15	IV, N = 9	V, N = 7	*p*-value
BOP, (Median, IQR)	22 (8, 48)	12 (8, 18)	30 (17, 53)	13 (10, 22)	33 (12, 47)	7 (4, 22)	0.0284
Total bacterial level (%)							
> 10^8^	36.8	38.5	35.3	60.0	33.3	42.9	0.75
< 10^8^	63.2	61.5	64.7	40.0	66.7	57.1	

Based on our previous observation that *S. cristatus* can inhibit the formation of *P. gingivalis* biofilm (Xie et al., 2000), we anticipated that a higher ratio of *S. cristatus* and *P. gingivalis* links to lower the risk for periodontitis development. The ratio of *S. cristatus* to *P. gingivalis* among the racial/ethnic groups was sequentially examined and compared. As shown in **Table 8**, only 40.9% of *P. gingivalis*-positive participants in the AA group had a ratio of higher than 100. On the other hand, 66.7% and 45.9% of *P. gingivalis*-positive participants in the CA and HA groups, respectively, had a ratio of higher than 100. Only 33.3% of *P. gingivalis*-positive participants in the CA group had a ratio of lower than 100, compared to 59.1% in the AA and 54.1% in HA groups. Although the percentage differences were not significantly different among the groups (*P*=0.17), it was clear that more participants in the AA group had the lower *S. cristatus*:*P. gingivalis* ratio and fared the worst in periodontal health. We are currently investigating a larger cohort of study participants in order to obtain statistically significant results.

**Table 8:** Correlation between *P. gingivalis* and *S. cristatus* in *P. gingivalis*-positive participants.

Ratio of bacterial levels	The ratios in the racial/ethnic groups (%, N)		
*S. cristatus*/ *P. gingivalis*	AA	CA	HA
<100	59.1 (13)	33.3 (7)	54.1 (20)
>100	40.9 (9)	66.7 (14)	45.9 (17)

## Discussion

To assess the potential risk factors for periodontitis development, we performed a comprehensive study to analyze the demographics, oral microbiological profiles, and clinical features of study subjects from different racial/ethnic backgrounds, all with an intact periodontium. Several factors that may contribute to periodontal health disparities were identified. Current models for the pathogenesis of periodontitis recognize *P. gingivalis* as a keystone pathogen that elevates the virulence of the entire microbial community (Hajishengallis et al., 2011; Darveau et al., 2012; Hajishengallis et al., 2012; Hajishengallis and Lamont, 2014). One key finding in this study is the differential detection rates of *P. gingivalis* among the AA, CA, and HA groups. *P. gingivalis* was detected in 23.5% of all participants; this percentage is similar to a previously reported rate of 25% (Griffen et al., 1998). However, the detection rate of *P. gingivalis* to be the highest in the HA group, when participants in specific groups were examined. Moreover, the detection rate of *P. gingivalis* in younger participants (aged 21–35 years) was higher in the AA and HA groups than in the CA group. More participants in the AA and HA groups exhibited higher levels of *P. gingivalis* in their dental plaques compared to the CA group. Our results suggest that the relatively high levels of *P. gingivalis* detected at younger ages may contribute to a higher risk for periodontitis in AA and HA populations. Our observations are in accordance with a previous analysis of 16S rRNA genes using deep sequencing that showed a higher abundance of *Bacteroidetes* and higher prevalence of *P. gingivalis* in AA than in CA subjects (Yang et al., 2019).

In the light of detection of *P. gingivalis* in dental plaques from both periodontitis patients and periodontally healthy subjects, it was suggested that different subsets of *P. gingivalis* strains exist in diseased and healthy periodontal sites (Griffen et al., 1998). An increasing body of evidence reveals that *P. gingivalis* strains with types II and IV FimA are often associated with periodontitis (Amano et al., 2000; van der Ploeg et al., 2004; Miura et al., 2005; Wang et al., 2020), while strains with type I *fimA* are associated with healthy periodontal tissues (Amano et al., 2000). In this study, the distribution of *P. gingivalis fimA* genotypes in the different racial/ethnic groups was compared. We found that the majority of *P. gingivalis*-positive individuals in the AA group carry the strain with type II *fimA*, which was significantly higher than that in the CA and HA groups. In addition, the type II and IV strains appeared to be associated with higher levels of BOP. These findings could explain a higher prevalence of periodontitis in the AA population (Amano et al., 1999; van der Ploeg et al., 2004; Miura et al., 2005; Wang et al., 2020). It is possible that *P. gingivalis* strains with *fimA* types II and IV in periodontally healthy subjects more likely break up a balanced host immune and induce uncontrolled inflammation in the periodontium (Yang et al., 2004).

We previously identified a negative correlation between *P. gingivalis* and *S. cristatus* in dental plaque samples from periodontitis patients (Wang et al., 2009). In the present study, higher ratios of *S. cristatus* to *P. gingivalis* in *P. gingivalis*-positive subjects in the CA group than those in the AA and HA groups with intact periodontium were also observed. It is reasonable to speculate that higher ratios of *S. cristatus* to *P. gingivalis* can keep *P. gingivalis* in check and inhibit the ability of the latter to elevate the virulence of the oral microbiota. In other words, the lower ratios found in the AA and the HA groups may render these individuals more susceptible to periodontitis.

It is well known that diabetes is a major risk factor for periodontitis (Preshaw et al., 2012). According to the CDC (https://www.cdc.gov), the incidence of new diabetes cases is higher among non-Hispanic AAs and people of Hispanic origin than among non-Hispanic Asians and non-Hispanic CAs. We did not observe an uneven distribution of diabetes cases among the different racial/ethnic groups in our study cohort of 340 participants. Smoking is another significant risk factor for periodontal disease development and progression (Zhang et al., 2019). According to the American Lung Association (https://www.lung.org/quit-smoking/smoking-facts/impact-of-tobacco-use/tobacco-use-racial-and-ethnic), adult smoking rates are 16.8% in AAs, 16.6% in CAs, and 10.1% in HAs, which are higher than what we observed in our cohort (9.2% in the AAs, 10.8% in the CAs, and 6.7% in the HAs). Although the smoking rate in the HA group appeared to be lower than that in the AA group and the CA group, the differences in percentages were not statistically significant. One plausible reason is that our sample size was not large enough to detect significant differences in diabetes incidence and smoking rate. In addition, participants in this study were recruited at dental clinic in an academic setting; hence, the cohort may not be representative of the general population. Future studies on larger and more representative patient cohorts are warranted. Moreover, our observation of zip code incomes agrees with a previously reported likelihood of an association between neighborhood socioeconomic circumstances and periodontitis (Borrell et al., 2006). However, further studies are needed to examine how neighborhood socioeconomic conditions impact the development of periodontitis and periodontal health disparities.

In this study, we collected dental plaque samples only once from the participants diagnosed as periodontal health or biofilm-induced gingivitis on an intact periodontium (without periodontitis) during their initial periodontal examination. The participants were not followed after the sample collection. Therefore, this study only provides preliminary information about the possible factors associated with periodontal health and further studies are needed in the future to discern their precise role in periodontitis development. Additionally, the present study was limited on *P. gingivalis* and several key oral bacteria. Whole bacterial profiles of the samples are under investigated using metagenome shotgun sequencing in our lab, which should provide clearer pictures on core microbiota associated with periodontal health disparities.

In conclusion, this work highlights the importance of differential oral microbiota in periodontal health disparities. Several risk factors may be linked to periodontal health disparities, including the presence of *P. gingivalis* at higher levels and at younger ages, the prevalence of specific subsets of *P. gingivalis* strains, and lower ratios of *S. cristatus*/*P. gingivalis*.

## Data availability statement

The original contributions presented in the study are included in the article/**Supplementary Material**. Further inquiries can be directed to the corresponding authors.

## Ethics statement

The studies involving human participants were reviewed and approved by the Committee for the Protection of Human Subjects of the University of Texas Health Science Center at Houston. The patients/participants provided their written informed consent to participate in this study.

## Author contributions

HX and B-YW conceived the study and supervised the project. AC and DW performed and verified the statistical analyses. B-YW, SS, and H-WM enrolled study participants. EG, JH, and B-YW collected medical and dental history data. M-HH and JH helped sample process. All authors contributed to the article and approved the submitted version.

## Supplementary material

Supplementary Table 1 Individual clinical data of the cohort.
